# Supplementary material for: Dual function of Zika virus NS2B-NS3 protease
Source: PLoS Pathog. 2023 Nov 27;19(11):e1011795. doi: 10.1371/journal.ppat.1011795 (PMC10723727; doi:10.1371/journal.ppat.1011795)
Supplement: S1 Fig — (A) Positions of cleavage sites for host and viral proteases at the junctions between individual viral proteins are indicated by arrows. (B) NS2B-NS3pro cleavage sequences in flaviviral polyproteins. Cleavage sites in the capsid protein C and at the NS2A/NS2B, NS2B/NS3, NS3/NS4A, NS4A/NS4B, and NS4B/NS5 boundaries are shown. ZIKV, Zika (GenBank AMB37295); WNV, West Nile virus (GenBank P06935); JEV, Japanese encephalitis (GenBank P19110); YFV, yellow fever (GenBank P19901); DENV1–4, dengue serotypes 1–4 (GenBank P33478, P29990, P27915, and P09866, respectively). (PDF) [file ppat.1011795.s001.pdf]

A

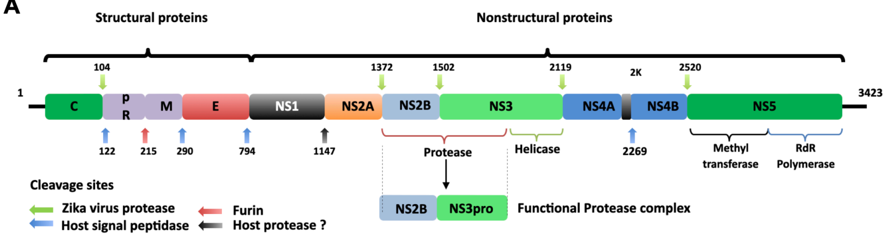

B

	Capsid C	NS2A/NS2B	NS2B/NS3	NS3/NS4A	NS4B/NS5
**ZIKV**	V^{125}TRR↓GSAY^{132}	S^{1369}GKR↓SWPP^{1376}	T^{1499}GKR↓SGAL^{1506}	A^{2116}GKR↓GAAF^{2123}	G^{2766}PRR↓PVKY^{2773}
**WNV**	Q^{102}KKR↓GGTA^{109}	N^{1367}RKR↓GWPA^{1374}	Y^{1498}TKR↓GGVL^{1505}	S^{2117}GKR↓SQIG^{2124}	G^{2522}LKR↓GGAK^{2529}
**DENV1**	R^{97}RKR↓SVTM^{104}	W^{1341}GRK↓SWPL^{1348}	K^{147}1KQR↓SGVL^{1478}	A^{2090}GRR↓SVSG^{2097}	G^{2489}GRR↓GTGA^{2496}
**DENV2**	R^{97}RRR↓SAGV^{104}	S^{1342}KKR↓SWPL^{1349}	K^{1472}KQR↓AGVL^{1479}	A^{2090}GRK↓SLTL^{2097}	N^{2488}TRR↓GTGN^{2495}
**DENV3**	K^{97}RKK↓TSLC^{104}	L^{1340}KRR↓SWPL^{1347}	Q^{1470}TQR↓SGVL^{1477}	A^{2089}GRK↓SIAL^{2096}	T^{2487}GKR↓GTGS^{2494}
**DENV4**	G^{96}RKR↓STIT^{103}	A^{1341}SRR↓SWPL^{1348}	K^{1471}TQR↓SGAL^{1478}	S^{2089}GRK↓SITL^{2096}	T^{2484}PRR↓GTGT^{2491}
**JEV**	Q^{102}NKR↓GGNE^{109}	N^{1370}KKR↓GWPA^{1377}	T^{1501}TKR↓GGVF^{1508}	A^{2120}GKR↓SAVS^{2127}	S^{2524}LKR↓GRPG^{2531}
**YFV**	R^{98}KRR↓SHDV^{105}	F^{1351}GRR↓SIPV^{1358}	G^{1481}ARR↓SGDV^{1488}	E^{2104}GRR↓GAAE^{2111}	T^{2503}GRR↓GSAN^{2510}

S1 Fig.
