# Supplementary figures and images for: Dual function of Zika virus NS2B-NS3 protease
Source: PLoS Pathog. 2023 Nov 27;19(11):e1011795. doi: 10.1371/journal.ppat.1011795 (PMC10723727; doi:10.1371/journal.ppat.1011795)

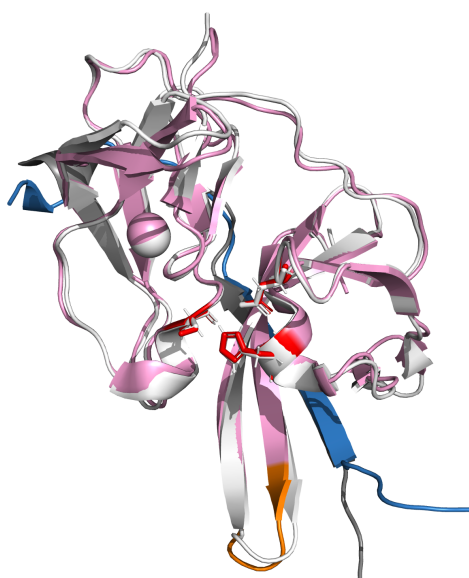

S2 Fig.

Supplement: S2 Fig — Color scheme: pink and blue—NS3pro and NS2B, respectively, from ZIKV. Light grey and dark grey—NS3pro and NS2B from JEV. Red color marks catalytic residues in ZIKV structure. Note a perfect overlap of all structurally resolved elements. The divergent tails of NS2B (blue and dark grey) were not resolved in either structure. (PDF) [file ppat.1011795.s002.pdf]

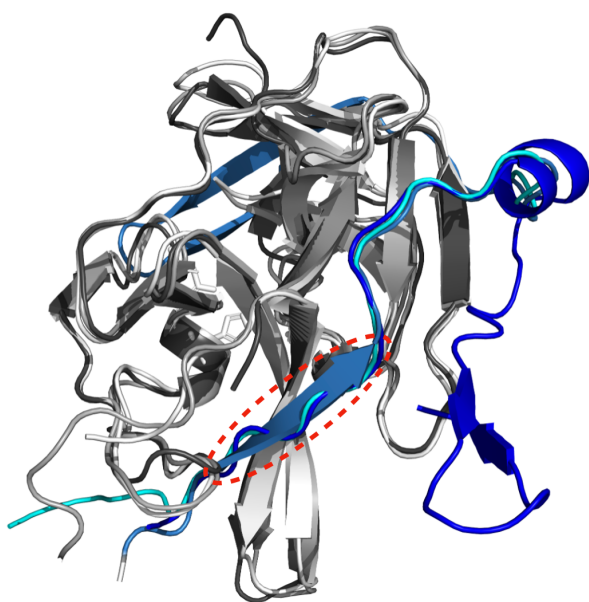

S3 Fig.

Supplement: S3 Fig — (A) NS2B-NS3pro constructs used in this study. All constructs were N-terminally fused with GST protein or HisTag for purification. NS2B central hydrophilic portion is shown in blue and the NS3 protease in green. L = GGGGSGGGG linker between NS2B and NS3pro. (B) Western blot analysis of purified wild-type and Mut7 NS2B-NS3pro proteins. (PDF) [file ppat.1011795.s003.pdf]

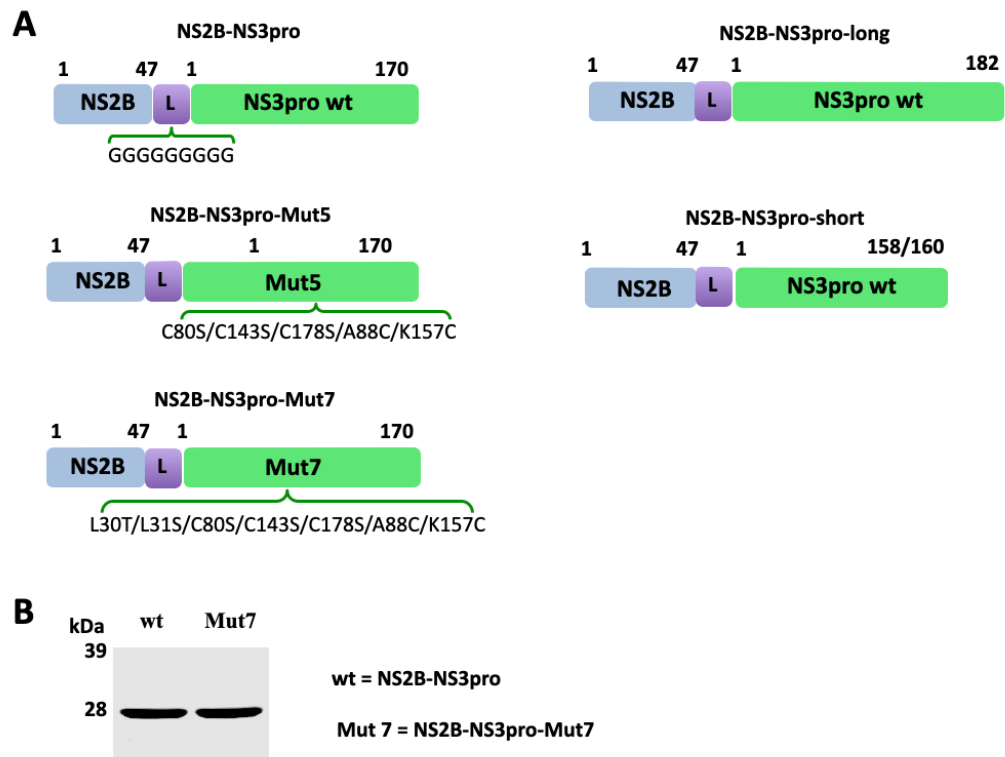

S4 Fig.

Supplement: S4 Fig — A. eZiPro (PDB ID GJ4), NS3pro and NS2B are marked with light and dark blue, respectively. Peptide fragment TGKR (bound to eZiPro) shown in green. B. bZiPro (PDB ID 5GPI), NS3pro and NS2B are marked with magenta and grey, respectively. Peptide fragment KKGE (bound to bZiPro) shown in orange. C. Superposition of A and B. Catalytic residues marked in red. Note that protease catalytic center is occupied in both crystal structures. (PDF) [file ppat.1011795.s004.pdf]

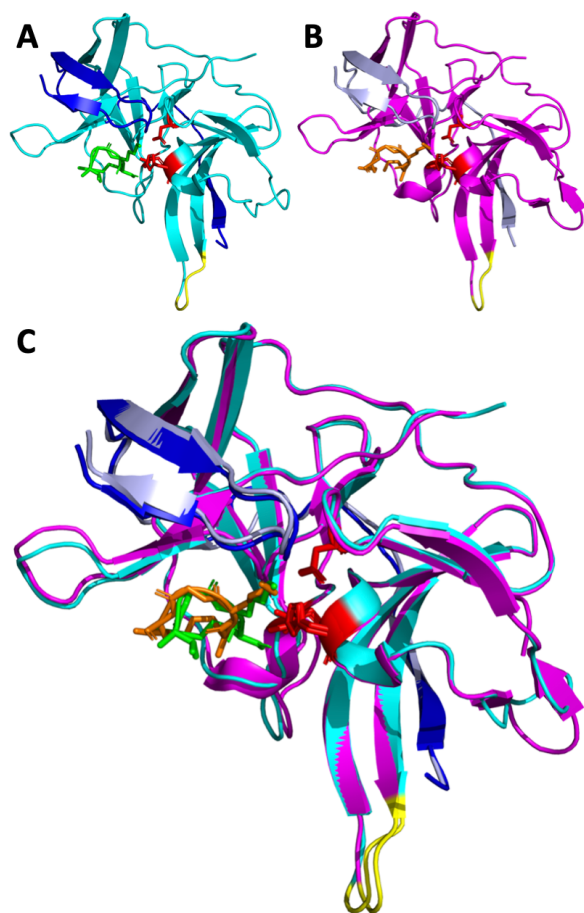

S5 Fig.

Supplement: S5 Fig — A. eZiPro (PDB ID GJ4), NS3pro and NS2B are marked with light and dark blue, respectively. Peptide fragment TGKR (bound to eZiPro) shown in green. B. bZiPro (PDB ID 5GPI), NS3pro and NS2B are marked with magenta and grey, respectively. Peptide fragment KKGE (bound to bZiPro) shown in orange. C. Superposition of A and B. Catalytic residues marked in red. Note that protease catalytic center is occupied in both crystal structures. (PDF) [file ppat.1011795.s005.pdf]
