# Supplementary material for: Dual function of Zika virus NS2B-NS3 protease
Source: PLoS Pathog. 2023 Nov 27;19(11):e1011795. doi: 10.1371/journal.ppat.1011795 (PMC10723727; doi:10.1371/journal.ppat.1011795)
Supplement: Attachment — Submitted filename: 133295_1_rebuttal_2696764_rvr34v.docx [file ppat.1011795.s006.docx]

**Manuscript number:** RC-2023-01821

**Corresponding author(s):** Alexey V. Terskikh

*[Please use this template only if the submitted manuscript should be considered by the affiliate journal as a full revision in response to the points raised by the reviewers.*

*If you wish to submit a preliminary revision with a revision plan, please use our "*[*Revision Plan*](https://docs.google.com/document/u/0/d/1HHp62Tfeb44nd3Gu_3ChB0b8gmMdEog7GjZcZFwHSiQ/edit)*" template.* ***It is important to use the appropriate template to clearly inform the editors of your intentions.****]*

## 1. General Statements [optional]

## 2. Point-by-point description of the revisions

3. **Reviewer #1** **(Evidence, reproducibility and clarity (Required)):**
4. *The current manuscript by Shiryaev et al describes their observation of the new function of zika NS2B-NS3 proteases. They have shown that NS2B-NS3 protease lacking the helicase domain binds to RNA and the interaction can be affected by protease inhibitors. Main two new findings are presented in the manuscript: super open conformation of the protease; RNA binding activity of the protease region. Although the manuscript is interesting, the design of the experiments is not convincing.*
6. Major issues:
7. *1. the claim of a super open confirmation is problematic. Using an artificial construct lacking the C-terminal portion of NS2B will of course generate the open conformation. This is a wrong definition unless you observe such a conformation in living cells.*

**Rebuttal Figure 1.** An overlap of ZIKV NS2B-NS3pro (PDB ID 7M1V) and JEV NS2B-NS3pro (PDB ID 4R8T). Color scheme: pink and blue - NS3pro and NS2B, respectively, from ZIKV. Light grey and dark grey - NS3pro and NS2B from JEV. Red color marks catalytic residues in ZIKV structure. Note a perfect overlap of all structurally resolved elements. The divergent tails of NS2B (blue and dark grey) were not resolved in either structure.

8. - We understand the skepticism towards a less known super-open confutation of flavivirus NS2B-NS3pro complex. In addition to our own structure of ZIKV NS2B-NS3pro (PDB ID 7M1V), the crystal structure of another orthologous flavivirus Japanese encephalitis virus (JEV) NS2B-NS3pro (PDB ID 4R8T) was discovered in 2015 ^1^. However, no functional analysis was provided for this crystal structure resulting in the lack of attention paid by the research community. We computed the overlay of the ZIKV NS2B-NS3 protease structures in the super-open conformation (PDB ID 7M1V, deposited by us in 2021) with the crystal structure of JEV protease (PDB ID 7M1V ) (**Rebuttal Figure 1**). We observed an almost identical organization of the critical NS3pro C-terminal loop between these two structures (RMSD 0.6A). Polypeptides with over 35% identity are very likely to have a similar fold^2^. Given over 50% identity(!) between flaviviral proteases across the family^3,4^, we posit that the super-open conformation demonstrated for JEV and ZIKV NS2B-NS3pro is a common feature of the Flaviviridae family. Further, NS2B peptide is always tightly associated with NS3pro via a three-strand beta-barrel (aa 49-58 of NS2B), which remains intact in all NS3Pro conformations. The C-terminal portion of NS2B progressively loses association with NS3pro, being mostly associated in the closed conformation, less so in the open, and even less in the super-open conformation. The G_4_SG_4_ linker between NS2B and NS3pro remains unstructured in all conformations. The native *C-terminal portion of NS2B (TGKR) is equally unstructured when competed out of the protease active site by another substrate.* It is unclear to us why “*lacking the C-terminal portion of NS2B will of course generate the open conformation”.*

**Rebuttal Figure 2**. Superimposition of eZiPro^5^ and bZiPro^6^ crystal structures. **A.** eZiPro (PDB ID GJ4), NS3pro and NS2B are marked with light and dark blue, respectively. Peptide fragment TGKR (bound to eZiPro) shown in green. **B.** bZiPro (PDB ID 5GPI), NS3pro and NS2B are marked with magenta and grey, respectively. Peptide fragment KKGE (bound to bZiPro) shown in orange. **C.** Superposition of A and B. Catalytic residues marked in red. Note that protease catalytic center is occupied in both crystal structures.

11. *2. It is odd that authors made homology model to generate open conformation structures. the authors did not cite the two papers of eZiPro (Phoo et al 2016 NC) and bZiPro (Zhang et al 2016, Science). these two structures show the closed conformation of protease in the absence and presence of a natural substrate.*
12. - We agree with the reviewer that in both constructs eZiPro^5^ and bZiPro^6^ of ZIKV NS2B-NS3pro are likely to exist in the closed conformation as documented by the crystal structures. However, in both cases, the active center of ZIKV NS2B-NS3pro is occupied with a short peptide fragment, which is sufficient to induce the closed conformation of NS2B-NS3 protease. We superimposed eZiPro (PDB ID 5GJ4) with bZiPro (PDB ID 5GPI) to better demonstrate that the active center in both structures is occupied either by tetrapeptide TGKR (T127-G128-K129-R130 ) originating from the NS2B C-terminus (eZiPro) or by a tetrapeptide KKGE (K14-K15-G16-E17) originating from a neighboring NS3 molecule (bZiPro) (**Rebuttal Figure 2**). Indeed, Zheng et al., 2016 ^6^ stated that: “the structure (bZiPro) does capture the protease in complex with a reverse peptide. The tetrapeptide K14K15G16E17 folds into a small hairpin loop to occupy the active site.” Further, Phoo et al., 2016 ^5^ stated that “binding of the ‘TGKR’ peptide to the catalytic site stabilizes the protease (eZiPro)”. To the best of our knowledge, so far there are no crystal structures of flaviviral NS2B-NS3 proteases in the closed conformation without peptide/inhibitor in the active center. We take it as a hint that the closed conformation is always induced by a substrate present in the active center.
13. Finally, we would like to draw the attention of this reviewer to the fact that the ^15^N R_2_ NMR signal from NS2B residues 65-85 is missing in bZiPro alone but re-appears when AcKR is added. This is consistent with the idea that without AcKR, bZiPro exists in the open conformation where much of the C-terminal part of NS2B is dissociated from NS3Pro and remains unstructured, thus resulting in the lack of NMR signal.
15. *3. RNA binding is novel, but is it observed in cells? only one method was used for testing the interactions, not other biophysical methods are used.*
16. - Given a complex network of protein-RNA interactions and the fact that NS3pro and NS3hel are connected by a single polypeptide, separating dynamically bound 11kB RNA to NS3pro from that to NS3hel in a native cell is a major technical challenge beyond the scope of this work. We employed a fluorescent polarization assay to demonstrate ssDNA and ssDNA binding to ZIKV NS2B-NS3pro. Subsequently, we employed a proteolytic activity assay with labeled peptide mimicking natural substrate for protease to demonstrate that the presence of ssRNA and ssDNA can efficiently inhibit proteolytic activity. To the best of our knowledge, this is the first indication that ssRNA or ssDNA could block proteolytic activity for any serine proteases, let alone a viral protease. Therefore, we consider the proteolytic activity assay used in the current work an orthogonal biochemical method supporting ssRNA binding to ZIKV NS2B-NS3pro.
17. *4. binding studies with RNA used artificial construct, how about the one with KTGR present like eZiPro. Keep in mind that the P1-P4 residues are present under native conditions.*

**Rebuttal Figure 3.** NS2B is always associated with NS3Pro via stable beta-barrel. Color scheme – various shades of grey represent NS3pro (white – closed, grey70 – open, grey30 – super-open. Corresponding NS2B – closed – skyblue, open – dark blue, super-open – cyan). The beta-barrel formed by all N-terminal fragments of NS2B and NS3pro outlined by a red dotted line. Noted progressive dissociation of SN2B from NS3pro in the open and super-open conformations.

18. **-** As mentioned by the reviewer, TGKR peptide was found in the active center in the eZiPro crystal. Indeed, the junction region between NS2B and NS3 protease contains native cleavage sites for the NS2B-NS3Pro and is naturally cleaved by protease during the viral polyprotein processing. However, the TGKR peptide representing P1-P4 positions will have to leave the active center after the cleavage to ensure enzyme processivity/cleaving additional targets (otherwise, the protease would get stacked after the first cleavage). Proteolytic activity assay utilizes the fluorogenic peptide labeled with FAM (such as TGKR-FAM; where FAM is a group representing P1’ position in this case). TGKR-FAM peptide will compete and easily replace cleaved TGKR peptide from the active center in proteolytic activity assay. In sum, the C-terminal end of NS2B will be competed out of the protease active center by the next substrate, and there is no evidence that it will be naturally placed back in the active center after each round of protease proteolytic activity. Indeed, several crystal structures of flaviviral NS2B-NS3Pro in open conformation lack the C-terminal part of NS2B in the active center. Our unpublished NMR studies demonstrated that the C-terminal part of NS2B is unstructured in solution if the substrate peptide or small molecule inhibitor are not present in the active center of the protease.
20. *5. authors built up nice models, it is great to consider the full length NS2B, but authors haven't taken into account the effect of NS2B on the open or closed conformation of the protease.*
21. **-** All crystal structures of flavivirus NS2B-NS3pro in the closed, open, or super-open conformations have NS2B associated withNS3pro via a beta-barrel (**Rebuttal Figure 3**), which is located at the opposite side from the RNA binding site. The transition from the closed to the open and to the super-open conformation is associated with the progressive dissociation of NS2B from NS3pro. Therefore, the effect of NS2B on NS3Pro is progressively diminished. In the closed conformation of NS3Pro, the negatively charged C-terminal part of NS2B is associated with the same positively charged grove as the RNA in the open conformation of NS3Pro. The C-terminal part of NS2B is dissociated from NS3Pro in the open conformation.
23. **Minor issues:**
24. *This manuscript shows the novel function of zika protease and conclude that protease binds to RNA. This is a novel finding, but the conclusion needs to be further confirmed, to avoid misinterpretations by future readers*
25. *6. closed, and super open conformations. But the definition was not carefully compared with current literatures. I am surprised that the two important papers are not cited. It is well known the G4SG4 linker affect the conformation of the protease.*
26. - The crystal structures and the proteolytic activities of gZiPro, eZiPro, and bZiPro are rather similar. In fact, K_m_ (μM) are 2.86 ± 0.90 for gZiPro, 6.332 ± 2.41 for bZiPro, and the IC 50s of BPTI inhibition for gZiPro, eZiPro and bZiPro are 350, 76 and 12 nM respectively. NS2B and NS3pro have a large binding area in the closed conformation. Upon changing the conformation to the open conformation (and even more so to the super-open conformation), the C-terminal part of NS2B is progressively dissociated from NS3Pro. Therefore, possible minor effects introduced by the G4SG4 linker is unlikely to affect any of the conclusions in our work.
28. *7. Authors need to show super open conformation is present in nature e.g. the model in which full length NS2B and NS3pro.*
29. - A full-length NS2B has 2 transmembrane domains, which tether the NS2B-NS3pro complex to the cell membrane (we have modeled the presence of such transmembrane domains to account for the orientation of NS2B-NS3pro with respect to the cell membrane). The full-length complex has never been crystallized or tested in any assay due to the major technical challenges associated with the modeling of complex transmembrane proteins.
31. *8. RNA is a charged molecule under some conditions, NS3 also have charged residues, it is important to show whether the binding between RNA-protease is relevant to the function*{Luo, 2010 #9270;Chernov, 2008 #9275;Xu, 2019 #10006}*, or is this due to the application of the artificial constructs used in this study. Why so many mutants are used?*
32. - The requirement of NS3pro for the helicase function was shown by several investigators ^7–9^. Given the structural independence of NS3pro and NS3hel, which mostly rules out the allosteric effect, RNA binding by NS3pro is a newly proposed function of NS3pro for the helicase activity. We demonstrated biochemically that RNA-bound to NS3pro inhibits its protease function. A variety of mutants were used to constrain the conformations of NS2B-NS3pro (e.g. enforce the super-open confirmation) for crystallization studies.
34. *9. Using a construct close to the native protease, at least the P1-P4 residues should be present. Using a peptide in the assay is also useful.*
35. - We were unable to interpret this critique.
37. *10. Test binding of RNA with protease using another method such as biophysical methods, or even gel shift assay*
38. - We thank the reviewer for this suggestion. Although the gel-shift assay seems to be a reasonable method to test the binding, given the ease of spontaneous conformational change (i.e. into the super-open conformation), this assay could result in a progressive loss of bound RNA during migration in the gel.
40. *11. I don't know the correlation between Figure 7 and Figure 6. The authors describe ploy A binding to protease, while Figure 7 is talking about Helicase binds to dsRNAs.*
41. - There is no correlation. Figure 6 describes the models for NS2B-NS3pro binding to ssRNA. Figure 7 describes a separate point, the direction of dsRNA processing by NS3hel.
43. *12. I am glad to see the consideration of full length NS2B, NS3 in the models Figure 8, 9 and 11, but there is no data to support any of the model proposed.*
44. - There is no experimental data. We have modeled the N-terminal and C-terminal parts of full NS2B, which are predicted to be inserted into the cell membrane due to their characteristic amphipathic helical structure.
46. *13. Is the linker a ploy G not G4SG4?*
47. The linker is GGGGSGGGG (G4SG4) as stated in Materials and Methods of the manuscript.
49. *14. Do the mutant sustain their protease activity?*
50. - All mutants with intact catalytic centers have protease activity, except the mutants with a disulfide bridge that fixes the polypeptides in the super-open conformation.
51. __________________________________________
53. **Reviewer #2 (Evidence, reproducibility and clarity (Required)):**
54. *The manuscript by Shiryaev et al., submitted to BioRXiv is an exploration of the ability of NS2B-NS3protease to bind RNA and its subsequent role in NS3 helicase processivity. The authors first utilize fluorescence polarization assays to demonstrate that NS2B-NS3protease can bind ssRNA with a strong affinity (and also ssDNA with lower affinity). They subsequently utilize mutational and small molecule inhibitor strategies in these assays to force the NS2B-NS3protease into different conformations, with the associated results inferring that the "open" conformation is responsible for ssRNA binding affinity. Furthermore, they demonstrate that ssRNA binding impairs protease activity, suggesting these roles may be exclusive in the viral life cycle. They also identified a number of small molecule ligands that target the putative ssRNA binding channel, and demonstrate that these ligands inhibit ssRNA binding by NS2B-NS3protease, providing potential inhibitor candidates for ZIKV. Finally, the authors utilized their crystal structures and others for the various conformations of NS2B-NS3protease to model ssRNA binding by the domain and the full NS3 protein, and used these models to propose a reverse inchworm model for NS3 travelling along ssRNA as it unwinds the dsRNA duplex. Overall, the authors utilize a comprehensive approach to demonstrate a number of novel findings (ssRNA binding by NS2B-NS3protease, small molecule ligands that inhibit this interaction) that would be of interest to both virologists and structural biologists. However, there are some important experimental design limitations and viral life cycle considerations that the authors should address before acceptance of the manuscript. Major and minor comments intended to improve the manuscript are outlined in more detail below.*
56. Major Comments:
57. *1. While the quantity of indirect data (ruled out closed and super-open, inhibitors of ssRNA binding pocket) suggest that the open conformation of NS2B-NS3protease is associated with ssRNA binding, the argument would be greatly strengthened by direct experimental data. Is there a mutational or small molecule approach to locking the NS2B-NS3 protease in the open conformation? If so, the authors should perform such experiments to strengthen the foundation of their argument.*
58. - Unfortunately, despite significant efforts, mutations or small molecules locking the NS2B-NS3 protease in the open conformation have not been identified for the ZIKV protease. However, several structures for NS2B-NS3 proteases have been documented in other flaviviruses (i.e., DENV PDB IDs 2FOM and 5T1V; WNV PDB ID 2GGV). Polypeptides with over 35% identity are very likely to have a similar fold^2^. Given over 50% identity(!) between flaviviral proteases across the family^3,4^, there is little doubt that ZIKV NS2-NS3 protease adopts an open conformation similar to all flaviviral proteases. Our modeling demonstrated that there are no sterically/structural problems in folding NS2B-NS3 protease into the open conformation.
60. *2. A negative control should be used in Figure 4A to strengthen the claim that ssRNA binding in the open conformation impairs protease activity (ie. include a curve for dsRNA). Such an experiment would lend support to ssRNA inhibition being due to specific binding instead of some other non-specific effect of increasing local nucleic acid concentration.*
61. - To address this critique, we have conducted the modeling of dsRNA binding to the open conformation of NS2B-NS3Pro. The model revealed that dsRNA could not be accommodated by the open conformation of the NS2B-NS3Pro complex (**Rebuttal Figure 4**). Indeed, dsRNA has a very different rigid structure compared to the extended form of the ssRNA chain. The dsRNA is unable to provide continuous interactions between negatively RNA backbone and positively charged side chain amino acids in NS3pro. The continuous interface on NS2B-NS3 protease interacting with ssRNA is an extension of the exit groove for one of the ssRNA strands exiting the NS3 Helicase after unwinding. Therefore, the ssRNA, but not dsRNA is naturally always present in close proximity of the NS2B-NS3Pro complex.

**Rebuttal Figure 4**. In silico modeling illustrating the dimensions of dsRNA structure that could not be accommodated by the open conformation of NS2B-NS3Pro.

63. *3. Due to the highly coupled roles of NS5 and NS3 in replication, the authors should include some more consideration of the role of NS5 in their complex. They very briefly address this interplay in the fifth paragraph of the discussion, but then neglect to discuss the implications any further. In particular (perhaps in a brief comparison to an NS3/NS5 modeling approach such as Brands et al., 2017; WIRES), the authors should consider some of the following questions: could the channel on protease domain lead to ssRNA entry site on RdRp?*
64. - Indeed, our model suggests that the negative strand (-)ssRNA exits from NS2B-NS3protease facing the ER membrane in the area where the protease is anchored to the ER membrane via the NS2B transmembrane domains. It is possible that NS3pro interacts with NS5 polymerase and “handles” (-)ssRNA to the NS5 polymerase. This scenario would modify Brands et al., 2017 model to add NS2B-NS3Pro complex between NS3Hel and NS5. However, at present, the NS3-NS5 (or NS2B-NS3-NS5) complex together has not been crystallized. It would be logical for NS5 polymerase to access the (-)ssRNA strand after it is released from NS2B-NS3Pro since the (-)ssRNA strands are used as a template for the (+)ssRNA which is used for polyprotein synthesis and packaging into viral particles.
66. *4. would NS5 interaction constrain or augment inchworm model of NS2B/NS3 translocation?*
67. - Yes, integrating NS5 interaction with the NS2B-NS3pro handling (-)ssRNA will augment the utility of the suggested reverse inchworm model.
68. *5. how does increased activity of NS3 when complexed with NS5 (Xu et al. 2019) align with proposed inchworm model?*
69. - We appreciate the reviewer's question. We think that NS2, NS3, NS4, and NS5 work in concert as one coordinated complex where various subunits of NS2 and NS4 may provide anchoring of the entire complex to the ER membrane. Indeed, such a complex has recently been proposed^6^. Also, see our response to the previous reviewer’s point (#4). We have incorporated this discussion into the revised manuscript.
70. Minor Comments:
    *1. Introduction, 4th paragraph, NS3-NS4 should read NS3-NS4A.*
71. - We corrected this sentence.
72. *2. Throughout the manuscript, the authors should denote some key amino acid residues in each figure to help orient the reader better to the observed structural changes and rotations. Inclusion, at least in the supplement, of the crystal structures of mutants solved herein should also be included.*
73. - We annotated the key residues in all figures (e.g. catalytic residues, loop interacting with the membrane, position of NS2B, and other elements) and kept the same orientation of complexes in all figures.
74. *3. Section: RNA binding inhibits the proteolytic activity of ZIKV NS2B-NS3pro, last sentence, NS2N-NS3pro should be NS2B-NS3pro*.
75. - We corrected this sentence.
76. *4. Section: Allosteric inhibitors of NS2B-NS3 protease interfere with RNA binding- first sentence: "The open conformation of NS2B-NS3pro is achieved by the rearrangement of NS2B cofactor (its dissociation from the C-terminal half of NS3pro) leading to a loss of proteolytic activity [32]. - the reference is not correct. I could not find the reference the authors refer to here and had not heard before that NS2B cofactor was able to disassociate from the C-terminal half of NS3pro; hence, this really needs to be appropriately referenced.*
77. - We have revised this sentence and added additional references. “The open conformation of NS2B-NS3pro is achieved by the rearrangement of NS2B cofactor (partial dissociation from NS3pro), leading to a loss of proteolytic activity^4,11^.”
78. *5. Section: Modeling RNA binding to ZIKV NS2B-NS3, first sentence - unwinds should be unwind*.
79. - We corrected this sentence.
81. *6. With respect to the results of Figure 3A, the authors should address that adding the linker alone to the NS3 protease may not be an accurate examination of its role/importance. The linker in this scenario is only constrained at its N-terminus, while it is always constrained at both termini during infection (and even more so by the interactions of those two linked domains [protease and helicase] with each other). As such, the authors statement that "observations suggests that the 12-aa linker region modulates RNA binding to NS2B-NS3pro" should be more strongly qualified to this effect. In addition, it would be interesting to see the effects of the linker mutations on ssRNA binding in the context of the full NS3 protein, albeit admittedly more complex due to the confounding ssRNA binding by the helicase domain.*
82. - We agree with this reviewer that the protease-helicase linker is also restrained at both termini. We have rephrased the statement in the revised manuscript. The goal of the experiment shown in Figure 3A was to examine whether a negatively charged linker is able to compete with ssRNA binding as we expected from the structural model. The mutational analysis of the protease helicase linker is, indeed, a very interesting subject that is, however, beyond the scope of this work.

    *7. The NS#hel should be changed to NS3hel in part (C) of figure legend for Figure 11*.
    - We corrected this mishap.
83. *8. The authors data in Figure 4A (and even more so the nature of the viral life cycle where 1000s of viral polyproteins are created from the first genome during infection) disputes the depiction in the inchworm model of how NS3 protease cleaves the polyprotein while the helicase binds ssRNA. At minimum, the authors need to discuss this discrepancy, and it is recommended that they modify the cartoon in their model to not include the ssRNA binding on the protease side of the equation (or show as alternative on that side to the existing cartoon).*
    - Indeed, as proposed by our reverse inchworm model, ssRNA is not bound to NS3Pro in the closed conformation, while NS2B-NS3pro has a protein substrate in the active center (Figure 11A). We agree that NS2B-NS3Pro in the closed conformation cannot bind ssRNA as we demonstrated in competitive cleavage assay. Only large amounts of ssRNA can shift the balance towards the open conformation which binds ssRNA. We think that most of the time NS2B-NS3Pro cycles between the open and the super conformations handling ssRNA (Figure 11(B-C_D), but as soon as protein substrate becomes available (typically a loop from a transmembrane viral polypeptide), NS2B-NS3Pro quickly switches to the closed proteolytically active conformation to act as protease.
84. *9. In the third paragraph of the discussion, the authors state "An alternative model of coupled transcription and translation where viral RNA is associated with ribosomes right after the release from NS2B-NS3 is also possible". Considering there is abundant evidence that translation and replication are exclusive and that translation does not take place in ROs, it would be prudent to remove such statements from the discussion. Without any supporting evidence, these statements will be misleading to readers by providing a false equivalency. The preceding discussion of RFs would be sufficient to contextualize your inchworm model in the broader viral life cycle (which was done quite well).*
85. - We have adjusted the discussion in the revised manuscript to avoid a false equivalency.

    *10. There were a number of aspects I appreciated about the manuscript and will briefly list a few here: 
    i) the focus on how different non-structural proteins effect the structure and function of 
    each other during the viral life cycle, which forms a more comprehensive and informative model 
    ii) the use of structural and functional assays as complementary approaches to studying the intra- and inter-protein relationships of NS3 
    iii) the depiction of the forks in Figure 10, which effectively demonstrated the channels and oriented the reader to the conservation data 
    iv) the use of small molecule inhibitors to modify structure and function of NS3, which greatly deepened the richness of the story from both a basic and applied science view point*
86. - We are very grateful to the Reviewer for these kind remarks.
87. *Reviewer #2 (Significance (Required)):
    Strengths and limitations: 
    - provides some experimental and modeling data to provide a new model for RNA interactions with the NS3pro-hel; may help inform models for enzyme function, mostly consistent with previous literature 
    - leaves out the NS5 RdRp, known to contribute to NS3 activity. 
    - some suggestions are made which might strengthen the conclusions and inclusions of additional controls would improve the data. 
    Advance 
    - conceptual, perhaps may provide some insight into mechanism; although limited by the lack of NS5 RdRp which is crucial to helicase activity. It is unclear if the ssRNA would be oriented this way given interactions with NS5 RdRp and MT domains (is the ssRNA routed to NS5 or* *along NS3, or potentially are both happening?) 
    Audience: 
    - quite specialist, but may include structural biologists and virologist alike. 
    Expertise of the reviewer(s): 
    - molecular virologists, RNA viruses - including flaviviruses; replication complex biogenesis, protein-RNA and RNA-RNA interactions. While comfortable with the concepts regarding complex formation, the appropriateness of computational modeling and RNA docking tools as well as structural biology is out of our area of expertise.* 
    __________________________________________________
92. **Reviewer #3 (Evidence, reproducibility and clarity (Required)):**

    *This paper investigates the nucleic acid binding properties of zika virus protease. In particular the data suggest that single stranded RNAs and DNAs are capable of binding to and inhibiting ZIKV protease at micromolar concentrations. With the use of active site inhibitors and mutants that lock the protease in closed and super-open conformation, the authors concluded that RNA binds to the open conformation. Through extensive modeling of the protease and helicase domains, this manuscript provides a model of how ssRNAs can bind to all conformations of the proteas, but the open conformation provides two positively charged forks that should be available to bind RNA.*
93. *SECTION A - Evidence, reproducibility, and clarity
    Major comments: 

    ·The main conclusions of this paper rely on the existence of the super-open conformation, however this conformation has not been reported in the scientific literature previously. Structures deposited in the pdb are referenced in this manuscript, however no citation for an accompanying publication is provided. This calls into question the biological relevance of this super open conformation. This is of particular concern because in other highly-homologous flaviviral proteases, structures that have been observed crystallographically (e.g. the open conformation of dengue virus protease) appear to be only very sparsely populated in solution. What is the evidence that the super-open conformation exists in solution.*
94. - Please, see our reply to question #1 from Reviewer 1.
95. *· The activity of each of the constructs used was not reported making it impossible to directly compare the impact of these changes on intrinsic activity. In particular, the NS2B-NS3 long construct is predicted to exist in the super-open conformation. If this is correct, it should show no activity against a peptide substrate.*
96. - We appreciate these concerns. The NS2B-NS3pro-long construct is proteolytically active (only NS2B-NS3pro-short construct is proteolytically inactive because its NS3pro C-terminal part is too short to fold into the closed conformation). It is unconstrained and likely capable of adopting all possible conformations (closed, open, super open). As we suspected, the negatively charged linker interferes with RNA binding, potentially via direct competition. Investigating the role of the protease-helicase linker is an exciting subject of a separate manuscript in preparation.
97. *· This paper reports that the IC50 is much weaker than the Kd for binding of ssRNA to ZIKV NS2B-NS3pro. Are orthogonal assays, such as thermal shift assay, available which could distinguish between the reported IC50 and the Kd.*
98. - Binding of ssRNA occurs in an area distinct from the protease active center. We think that there is a constant competition between C-terminal NS2B binding/release versus ssRNA binding/release from NS3pro. We think that ssRNA “catches” the moment when protease has the open conformation and freezes that conformation by blocking the C-terminal of NS2B from binding to NS3Pro. In terms of thermal shift assay, the structure of NS3Pro is changed, only the C-terminal of NS2B is affected. Note that the ^15^N R_2_ NMR signal from NS2B residues 65-85 is missing in bZiPro alone but re-appears when AcKR is added^6^. This is consistent with the idea that without AcKR, bZiPro exists in the open conformation where much of the C-terminal part of NS2B is dissociated from NS3Pro and remains unstructured, thus resulting in the lack of NMR signal. Taken together, these observations suggest that thermal shift assay is unlikely to be of much help.
99. *·This paper suggests that ssRNA binds to the open conformation of ZIKV NS2B-NS3pro, however no experimental evidence, only modeling has been used to suggest binding to the open conformation. In Dengue virus protease, the M84P variant has been reported to lock the protease into the open conformation. How does the F84P variant of ZIKV NS2B-NS3pro impact ssRNA binding?*
100. - We appreciate this question. Indeed, M84P mutation shifts Dengue NS3Pro to the open conformation, which is proteolytically inactive^12^, consistent with our reverse inchworm model. We have not investigated the effect of this mutation on ZIKV NS3pro. We expect this mutation has a similar effect in ZIKV NS3pro in Dengue NS3Pro.
101. *·The relevance of the discussion on the co-crystallization of NSC86314 with the Mut7was not clear. What point was being made?*
102. - We provide a proof-of-principle for a novel class of allosteric inhibitors that specifically target newly identified druggable pockets present in the open and super-open conformations of ZIKV NS2B-NS3pro. Our results suggest that such allosteric inhibitors can interfere with the RNA-binding activities of NS2B-NS3pro in addition to blocking the protease activity. The co-crystallization of NSC86314 with the Mut7 confirms a novel pocked bound by NSC86314.
103. *- These data show that both active site and allosteric inhibitors block binding of ssRNA to the protease. The paper also suggests that ssRNA only binds to the open conformation. What is the evidence that the allosteric inhibitors do not enable or promote formation of the open conformation?*
104. - We thank this reviewer for an interesting question. Indeed, we have no evidence of whether allosteric inhibitors enable or promote the formation of the open conformation. This is formally possible and will need to be investigated.
105. *· This paper makes two claims about the function of the protease. The title should specify what those dual functions are (proteolytic activity and ssRNA-recruitment).*
106. - We appreciate this reviewer's suggestions for the title.
107. *· The discussion of Figures 6 and 9 are highly similar. The main takeaway points for both figures seem to be nearly identical: the presence of two positively charged pitchfork on the open conformation. The distinction between these two figures should be more significantly and explicitly stated.*
108. - Figure 6 presents several models that provide evidence for the open conformation of ZIKV NS2B-NS3pro being uniquely suitable to bind RNA. Figure 9 presents several models of the entire RNA-NS2B-NS3pro-NS3hel complex anchored into the ER membrane. Figure 9 illustrates that the open conformation of NS2B-NS3pro provides two positively charged/polar forks, contiguous with the positively charged groove on NS3hel. Figure 6 does not illustrate that point.
110. *- Mention explicitly in the materials and methods if the 12-amino acid linker is present in all the mutants used.*
111. - This is mentioned explicitly and shown in Supplementary Figure 2A.

     *Minor comments: 
     · Figure 1. The rotation that promotes the transitions from orientation in panel A to that in panel B should be drawn. 
     · FAM should be defined in the legend of Figure 2. 
     · The term Cold should be changed to unlabeled. 
     · Please check labels for the supplementary Figure 2. For example one label states 1-1 but it 
     should be 1-170. 
     · Figure 1C does not exist and it is referenced in the results section under "NS2B-NS3pro substrate-mimicking inhibitors compete with RNA binding." 
     · As discussed above, if the super open conformation is going to be addressed in this paper, then either a reference for the manuscript describing those structures should be included, or this manuscript should include in the materials and methods the procedure on crystallization, data collection, structure determination, refinement, and analysis as well as a table for crystallographic data and refinement statistics. 
     · Adjust figure arrangement (ABCED to ABCDE) in Figure 11.*
112. - We thank this reviewer for all minor comments. We corrected the above-mentioned errors in the manuscript.

     *Reviewer #3 (Significance (Required)): 
     It is well established that the flaviviral proteases exist in different conformations but most of the structures published are concentrated on the closed conformation which is the one required for effective substrate processing. The open conformation has recently been the subject of increased interest, especially with the discovery of allosteric inhibitors for which modeling suggests that these compounds result in the dissociation of the C-terminal region of NS2B from the NS3. This paper adds important insights into the function of the open conformation and in general implicitly shows the importance of the dynamic nature of ZIKV NS2B-NS3pro. In addition to these insights, this paper aptly demonstrates that ssRNA can bind and inhibit these proteases as has not been shown previously. 
     I am a senior graduate student working on characterizing and understanding the mechanism of action of allosteric compounds against viral proteases, specifically proteases from Zika and dengue viruses.*
113. **References.**
115. 1.	Weinert T, Olieric V, Waltersperger S, Panepucci E, Chen L, Zhang H, Zhou D, Rose J, Ebihara A, Kuramitsu S, Li D, Howe N, Schnapp G, Pautsch A, Bargsten K, Prota AE, Surana P, Kottur J, Nair DT, Basilico F, Cecatiello V, Pasqualato S, Boland A, Weichenrieder O, Wang BC, Steinmetz MO, Caffrey M, Wang M. Fast native-SAD phasing for routine macromolecular structure determination. Nat Methods. nature.com; 2015 Feb;12(2):131–133. PMID: 25506719
116. 2.	Solis AD, Rackovsky SR. Fold homology detection using sequence fragment composition profiles of proteins. Proteins. 2010 Oct;78(13):2745–2756. PMCID: PMC2933786
117. 3.	Brinkworth RI, Fairlie DP, Leung D, Young PR. Homology model of the dengue 2 virus NS3 protease: putative interactions with both substrate and NS2B cofactor. J Gen Virol. 1999 May;80 ( Pt 5):1167–1177. PMID: 10355763
118. 4.	Aleshin AE, Shiryaev SA, Strongin AY, Liddington RC. Structural evidence for regulation and specificity of flaviviral proteases and evolution of the Flaviviridae fold. Protein Sci. 2007 May;16(5):795–806. PMCID: PMC2206648
119. 5.	Phoo WW, Li Y, Zhang Z, Lee MY, Loh YR, Tan YB, Ng EY, Lescar J, Kang C, Luo D. Structure of the NS2B-NS3 protease from Zika virus after self-cleavage. Nat Commun. 2016 Nov 15;7:13410. PMCID: PMC5116066
120. 6.	Zhang Z, Li Y, Loh YR, Phoo WW, Hung AW, Kang C, Luo D. Crystal structure of unlinked NS2B-NS3 protease from Zika virus. Science. science.org; 2016 Dec 23;354(6319):1597–1600. PMID: 27940580
121. 7.	Luo D, Wei N, Doan DN, Paradkar PN, Chong Y, Davidson AD, Kotaka M, Lescar J, Vasudevan SG. Flexibility between the protease and helicase domains of the dengue virus NS3 protein conferred by the linker region and its functional implications. J Biol Chem. 2010 Jun 11;285(24):18817–18827. PMCID: PMC2881804
122. 8.	Chernov AV, Shiryaev SA, Aleshin AE, Ratnikov BI, Smith JW, Liddington RC, Strongin AY. The two-component NS2B-NS3 proteinase represses DNA unwinding activity of the West Nile virus NS3 helicase. J Biol Chem. 2008 Jun 20;283(25):17270–17278. PMCID: PMC2427327
123. 9.	Xu S, Ci Y, Wang L, Yang Y, Zhang L, Xu C, Qin C, Shi L. Zika virus NS3 is a canonical RNA helicase stimulated by NS5 RNA polymerase. Nucleic Acids Res. 2019 Sep 19;47(16):8693–8707. PMCID: PMC6895266
124. 10.	Klema VJ, Padmanabhan R, Choi KH. Flaviviral Replication Complex: Coordination between RNA Synthesis and 5’-RNA Capping. Viruses. 2015 Aug 13;7(8):4640–4656. PMCID: PMC4576198
125. 11.	Shiryaev SA, Aleshin AE, Muranaka N, Kukreja M, Routenberg DA, Remacle AG, Liddington RC, Cieplak P, Kozlov IA, Strongin AY. Structural and functional diversity of metalloproteinases encoded by the Bacteroides fragilis pathogenicity island. FEBS J. 2014 Jun;281(11):2487–2502. PMCID: PMC4047133
126. 12.	Lee WHK, Liu W, Fan JS, Yang D. Dengue virus protease activity modulated by dynamics of protease cofactor. Biophys J. 2021 Jun 15;120(12):2444–2453. PMCID: PMC8390872
